# Review of recently used techniques and materials to improve the efficiency of orally administered proteins/peptides

**DOI:** 10.1007/s40199-019-00316-w

**Published:** 2019-12-06

**Authors:** Yousif H-E. Y. Ibrahim, Géza Regdon, Elnazeer I. Hamedelniel, Tamás Sovány

**Affiliations:** 1 Institute of Pharmaceutical Technology and Regulatory Affairs, University of Szeged, Eötvös u. 6, Szeged, H-6720 Hungary; 2 Pharmaceutics Department, Omdurman Islamic University, Omdurman, Sudan

**Keywords:** Biopharmaceuticals, Enzyme inhibitors, Permeation enhancers, Mucoadhesive

## Abstract

**Objectives:**

The main objective of present review is to explore and evaluate the effectiveness of recently developed methods to improve the bioavailability of orally administered biopharmaceutical drugs.

**Methods:**

A systematic search of sciencedirect, tandfonline and Google Scholar databases based on various sets of keywords was performed. All results were evaluated based on their abstracts, and irrelevant studies were neglected during further evaluation.

**Results:**

At present, biopharmaceuticals are used as injectable therapies as they are not absorbed adequately from the different routes of drug administration, particularly the oral one. Their insufficient absorption is attributed to their high molecular weight, degradation by proteolytic enzymes, high hydrophilicity and rigidity of the absorptive tissues. From industrial aspect incorporation of enzyme inhibitors (EIs) and permeation enhancers (PEs) and mucoadhesive polymers into conventional dosage forms may be the easiest way of formulation of orally administered macromolecular drugs, but the effectiveness of protection and absorption enhancement here is the most questionable. Conjugation may be problematic from regulatory aspect. Encapsulation into lipid-based vesicles sufficiently protects the incorporated macromolecule and improves intestinal uptake but have considerable stability issues. In contrast, polymeric nanocarriers may provide good stability but provides lower internalization efficacy in comparison with the lipid-based carriers.

**Conclusion:**

It can be concluded that the combination of the advantages of mucoadhesive polymeric and lid-based carriers in hybrid lipid/polymer nanoparticles may result in improved absorption and might represent a potential means for the oral administration of therapeutic proteins in the near future.

**Graphical abstract:**
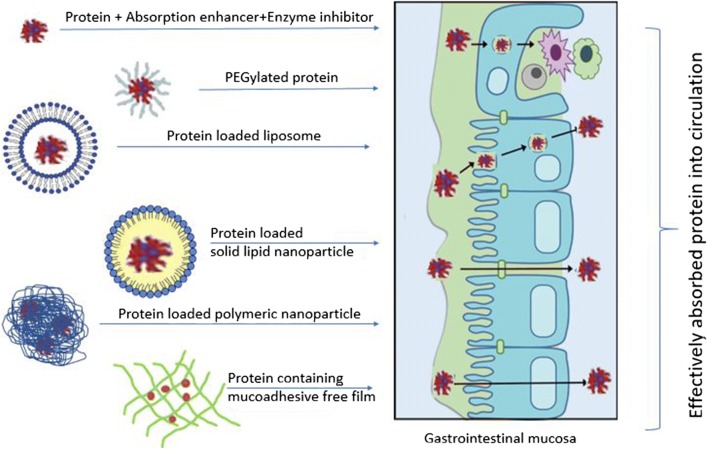
Delivery systems for oral protein daministration

## Introduction

Various diseases like diabetes, malignant tumors and some types of infections have been managed by peptides and proteins. In addition, peptides and peptidomimetics can serve as immunomodulating agents [1]. They produce their response either by antigenic properties or by stimulating the immune system as an agonist. Some intensively investigated peptides, such as cyclosporine, tuftsin, muramyl dipeptide (MDP) and thymic peptide analogues have already been used as therapeutic peptides [2]. Host defense peptides (HDPs) were accepted on a large scale as immune system stimulators and modulators, their effects include wound healing and the induction of both intra- and extracellular bactericidal effect through phagocytosis [3]. Consequently, biopharmaceuticals, including hormones, enzymes and immunomodulators, play an important role through the controlling of various functions, therefore they are useful in clinical practice to treat or prevent human disorders pathophysiological processes [4]. These days various macromolecules are intensively examined and more than thirty have been accepted by the Food and Drug Administration (FDA) for commercialization [5].

The design, formulation and peroral administration of therapeutically active biomolecules have represented a difficulty as well as a target for several years, and until now only a few biopharmaceuticals (insulin derivatives, interferon alpha, calcitonin, growth hormone, etc.) are known to be in clinical development [6, 7], and even less macromolecule is commercialized currently for oral administration (Table 1).

**Table 1:** Macromolecules commercialized for oral administration

Trade name	Drug	Company
Leftose	Lysozyme	Wellchem
Linzess	Linaclotide	Allergan
Trulance	Plecanatide	Synergy Pharmaceuticals

As a class, biopharmaceutical drugs, such as proteins and peptides, have the advantages of higher potency and specificity compared to small molecular drugs. These advantages are related to their rigid and complex structure, which at the same time represents the greatest obstacle in designing and formulating an oral delivery system of these macromolecules [8]. Accordingly, in the past significant interest was focused on the delivery of oral macromolecules in the hope of controlling different diseases and achieving better patient compliance by employing advanced pharmaceutical biotechnology for production and development [9]. Recently, the formulation of polymers with mucoadhesive properties as intestinal patches containing safe surfactant, as an oral insulin delivery system, has been one of the most studied techniques [10]. Biocompatible and biodegradable polymeric nanocarriers and lipid based nanoparticles have also come forth as promising oral delivery platforms for these biopharmaceuticals, as these systems give protection against proteases as well as control the release of proteins [11, 12]. The increasing importance of proteins/peptides can be explained as a result of three main developments: evolution in the analytical methods, which has promoted the discovery of a huge number of peptides and hormones applicable as biopharmaceuticals; good knowledge about the role of these molecules in the regulation of human pathophysiology; and the development of biotechnology and genetic engineering, which enables the production of biomolecules in a bulk quantities [13].

## Barriers to oral absorption

Any orally administered drug will face many barriers while passing along the gastrointestinal tract (GIT) before reaching the targeted absorptive capillaries at the absorption site of the sub-epithelial tissue. The most frequently encountered barriers are stomach acidity and the intestinal milieu, the tight junctions (TJs), which prevent the paracellular way, the external cells of the GIT and finally the subepithelial tissues [14]. The epithelium layer of the intestinal tract is a group of consolidated cells which act as a cover for the GIT and as mucosal immunological defense against the invading pathogens and harmful chemicals. The most common absorptive areas throughout the intestine are the microvilli covered apical surfaces of enterocytes, which are negatively charged. The distance between microvilli is around 25 nm, thus they prevent the passage of larger molecules [15]. Therefore, these cells act as a physical-, whereas the degrading enzymes represent a biochemical barrier. Therefore the understanding of the these barrier mechanisms and finding the way to overcome the limitations of macromolecule transportation are essential to develop effective oral protein/peptide delivery systems [16, 17].

**Fig. 1:**
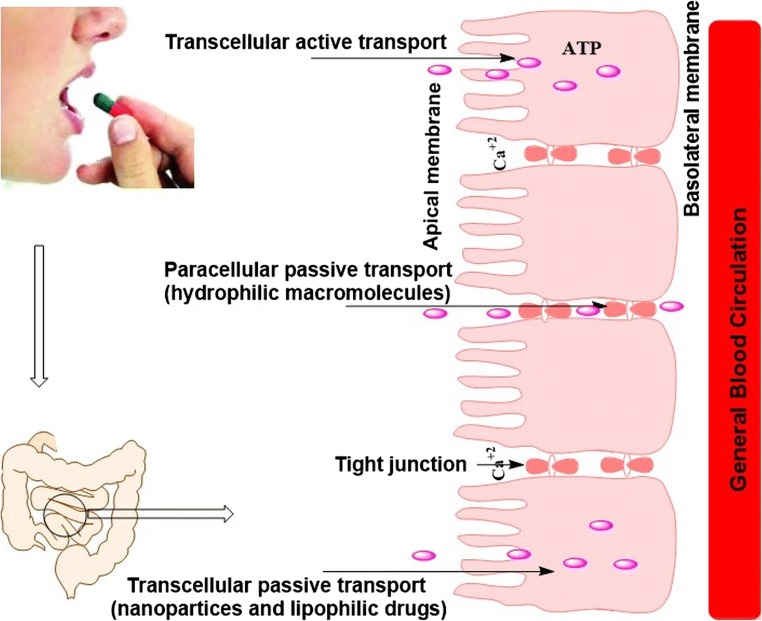
General pathways of absorption across the small intestines

The crossing of the cell barrier is possible via various ways; passively through diffusion, crossing the hydrophobic TJs or transepithelial cells, transcellularly via facilitated transport and by carrier-mediated transport (Fig. 1) [18]. Moreover, the absorption of both some biomolecules and some drugs may vary along the various parts of the GIT due to variation in the pH values, surface areas, activity of proteases and permeability of the absorptive site. Therefore, the determination of the proper region of the GIT for the chosen peptide/protein will be the primary step in the design and development of an oral dosage form with improved bioavailability [19].

### Intestinal digestive environment

The greatest difficulty encountered in the case of orally administered bioactive macromolecules is the lumen of the small intestine, where there is high concentration of proteolytic enzymes secreted by the pancreas and by mucosal cells. Another main enzymatic obstacle is the border of the epithelial cells, which contains around fifteen degrading enzymes with high selectivity for the breakdown of the macromolecular biomolecules [20]. In addition, the colon contains various enzymes produced by the local microflora, which should also be taken into account [21]. Generally, the degradation of administered biomolecules depends on numerous mechanisms adopted by these enzymes, and the overall result is that the byproducts of macromolecule degradation, such as short peptide chains and amino acids, have no ability to produce the required effect [22].

### Tight junction (TJ)

As mentioned above, drugs may penetrate across the membranes through the following pathways: paracellular pathway, transcellular pathway and through transport via microfold cells. Recently, some researchers have investigated the applicability of the absorption of drug entities from the small intestines via receptor-mediated, clathrin-mediated and even caveolae-mediated endocytosis. Most drugs are transported transcellularly, but for hydrophilic molecules (like proteins and peptides) paracellular absorption is the main pathway. However, this gate is tightly closed by tight junctions (TJs) [23, 24]. TJ proteins are associated with higher paracellular permeability, which is highly explicit throughout the small intestines [25]. TJs contain four types of unique transmembrane proteins: occludin, claudins, junctional adhesion molecules (JAMs) and tricellulin [26]. TJs are not static protein structures, they serve as penetration regulators of the molecules across the intestinal epithelium. Some penetration enhancers have the ability to loosen the TJs and thus facilitate the paracellular absorption of drug molecules [27].

### Mucous barrier

The mucosa is covered by the mixture of mucins, ions and proteins; therefore, it is a rigid layer which acts as a coat to the intestinal lumen and is bound to the surface by a glycoprotein structure (about 500-nm thick). The primary role of the mucosal layer is the regulation of pH at the lumen surface and thus results in the formation of an acidic microenvironment [28]. The mucosal layer has different thickness and turnover values regarding the anatomical position, pathophysiological status and interaction with the external environment [29]. Generally, the mucous layer acts as a physical barrier as a result of its negative charges and lipophilic nature, whereas the general hydrophilicity of mucus also acts as an interactive barrier, which retards the movement of the molecules within and through the mucus. The dynamic behavior of the mucosal layer is due to its continuous secretion and sloughing from the surface of the mucosal membrane, therefore mucus represents a rigid gel barrier to drug delivery [30].

## Formulation aids and techniques for improving bioavailability

Due to limiting factors such as large molecular weight, hydrophilic nature, inactivation due to stomach secretions and intestinal proteases, first pass effect, and tendency to aggregation, the bioavailability of orally administered proteins/peptides is usually recorded less than 1% [31]. Numerous approaches have been taken by researchers to improve the oral delivery of therapeutic proteins, like insulin. The most studied strategies include the use of permeation improvers, protease inhibitors, mucoadhesive polymers, polymeric nanoparticles, liposomal encapsulation, modification of the structure and microsphere encapsulation [7, 24].

### Permeation enhancers approach (PEs)

The use of permeation enhancers represents the most common approach of protein delivery, their use can modulate the characteristics of the absorptive epithelium and may facilitate both transcellular or paracellular absorption. Therefore, it is an applicable strategy to enhance the bioavailability of administered macromolecules [32]. These agents were first investigated twenty years ago to enhance the absorption of pharmacologically active molecules with poor bioavailability due to their low permeability as well as in an attempt to develop non-injectable systems for insulin delivery [33]. The enhancing effect through the paracellular pathway is due to the opening of TJs, whereas the transcellular pathway ensues from the increased permeability of the plasma membrane. Both pathways may be possible for one enhancer, but the number of enhancers that increase transcellular membrane permeability is 10 times higher than the number of those increasing paracellular absorption [34]. Calcium chelators act by stirring the cells through calcium depletion, which results in loosening the attachments of the TJs. In contrast, surfactants work through the disruption of the barrier function of the epithelium [35]. The less damaging paracellular pathway by a transient opening of TJs seems to be more rational and safer when compared to the disruption of the cell membrane structure. Nevertheless, the successful improvement of oral bioavailability in vivo necessitates the concurrent delivery of the drug and efficient concentrations of the absorption promoter to the intended absorption site [36]. It is also notable that the effectiveness of absorption enhancers is not the same along the GI tract due to the variations of numerous parameters, such as membrane thickness, morphology of the cells, proteolytic activity, lipid composition and fundamental protein interactions [37]. Moreover, despite the effective promotion of the oral absorption of poorly absorbable molecules, the use of PEs should be evaluated carefully as they can cause non-specific absorption and they must be avoided in the case of patients suffering from irritable bowel disease, celiac disease and inflammatory bowel disease [38]. Therefore, PEs offer the greatest potential when incorporated in localized delivery systems, like hydrogels and intestinal patches, to avoid non-specific absorption [39].

The general classes of PEs are demonstrated below in Table 2.

**Table 2:** Classification of permeation enhancers [33, 40–49]

Class	Example	Mechanism and pathway
Surfactants	polysorbates, poloxamer 407, Tween 80, labrasol, sodium dodecyl sulphate, lauryl methyl glucamide	inhibiting the effect of P-glycoprotein and modulating TJs, transcellular and paracellular pathways
Chitosan derivatives	Di- and tri-methyl chitosan, carboxymethyl chitosan	Strong mucoadhesion, opening tight junctions, maily paracellular pathway
Multicarboxylic acids	Citric acid, ethylenediaminetetraacetic acid (EDTA)	Chelating the calcium ions at the absorptive tissues and loosening the TJs, mainly paracellular pathway
Bile acid salts	Sodium cholate, glycocholate, taurocholate and deoxycholate	Enhancing lymphatic uptake or modulating TJs, both transcellular and paracellular pathways
Fatty acids and fatty alcohols	Stearic acid, octanoic acid, palmityl alcohol	

Besides the promotion of the transport of small drug molecules, sodium salicylate and EDTA have also demonstrated an improved oral bioavailability of insulin in dogs and rabbits [50]. They increase the paracellular transport of drug entities through affecting the permeability of TJs by chelating the membrane-bound calcium ions [51]. Medium chain fatty acids (as shown in Table 1) and gel-forming polymer media for example, octreotide may be also utilized to improve the efficiency of orally administered macromolecules [33]. Chitosan is a positively charged polymer commonly considered as an effective and harmless penetration enhancer for therapeutic macromolecules along the intestinal lumen via a reversible integrity modulation of epithelial TJs in a concentration dependent manner [52–54]. Phenyl piperazine at a concentration of 0.1%*w*/w has also been considered as a safe and effective transepithelial permeation enhancer amongst fifty-one studied promoters from eleven discrete chemical categories [39]. Sodium dodecyl sulfate (SDS) has also been reported as an effective, potent and safe absorption enhancer in oral formulations, as neither the change the epithelial surface, nor toxic luminal absorption has been reported [55]. Bile salts may serve as effective aids in drug formulation since they may improve absorption through both transcellular and intercellular paths. An investigation has shown the enhancement of heparin absorption by either the chemical conjugation of heparin or physical mixing with bile acids [55–57].

### Enzyme inhibitors (EIs) approach

One of the key issues to achieve appropriate oral activity is to protect the therapeutic peptides against luminal breakdown caused by the presence of various proteases. The inhibition of these proteolytic enzymes is achieved mainly by two mechanisms: local modulation of the pH away from the optimum ranges of peptidases or binding to target enzymes and limiting their activity [58]. In recent studies the use of numerous trypsin and α-chymotrypsin inhibitors have been investigated, such as soybean trypsin inhibitor, camostat mesylate, pancreatic inhibitor, amastatin, bestatin, aprotinin, boroleucine, bestatin, and aminopeptidase inhibitors, such as puromycin, to control the effect of these enzymes [16, 59]. The coadministration of oral insulin and EIs resulted in an improved hypoglycemic effect, which may be explained either by protecting insulin from the degradation activity of proteases or enhancing the absorption of insulin, or both at the same time [60]. Similarly, the concurrent administration of insulin microcrystals with protease inhibitor resulted in improved bioavailability also in the case of pulmonary delivery, and the absorption enhancement was the highest with soybean trypsin inhibitor among all the tested inhibitors [61]. Duck and chicken ovomucoids (DkOVM and CkOVM) have been reported as a unique class of protease inhibitors. Dissolution stability investigations showed that the percentage of insulin remaining for absorption increased dramatically against the action of proteases (trypsin and chymotrypsin) when administered with CkOVM and DkOVM [62].

As it was mentioned above, another technique to inhibit enzyme activity is to modify the pH at the targeted absorption site as the activity of proteases is extremely sensitive to pH change; intestinal proteases are active at a relatively elevated pH, thus lowering the pH at this site may decrease the activity of the enzymes present [63]. A pH modulator like citric acid (CA) can be utilized to suppress lumen peptidases, and it has been reported to be a helpful excipient for the oral delivery of some peptides, as the proteolytic action is particularly elevated in the upper part of the intestines [58]. Nevertheless, safety issues should be taken into consideration for formulations that contain any kind of protease inhibitors as these agents may interact with dietary proteins or rupture the integrity of the mucosal surfaceand cause upregulated enzyme secretion after long-term treatment [64, 65]. Moreover, the use of enzyme inhibitors may increase the amount of the intact drug at the absorption site but will not help passing through biological membranes. Therefore, the combination of the various approaches may be essential to have an appropriate therapeutic effect. In a recent study, enteric-coated capsules have been developed for oral insulin delivery, consisting of a greasy mixture of omega-3 fatty acids, containing insulin, EI such as aprotinin and chelating agent or bile acid salt as PE acid. This formulation has passed Phase II-a of clinical trials and is progressing into Phase II-b [66].

### Bioadhesive polymer approach

**Fig. 2:**
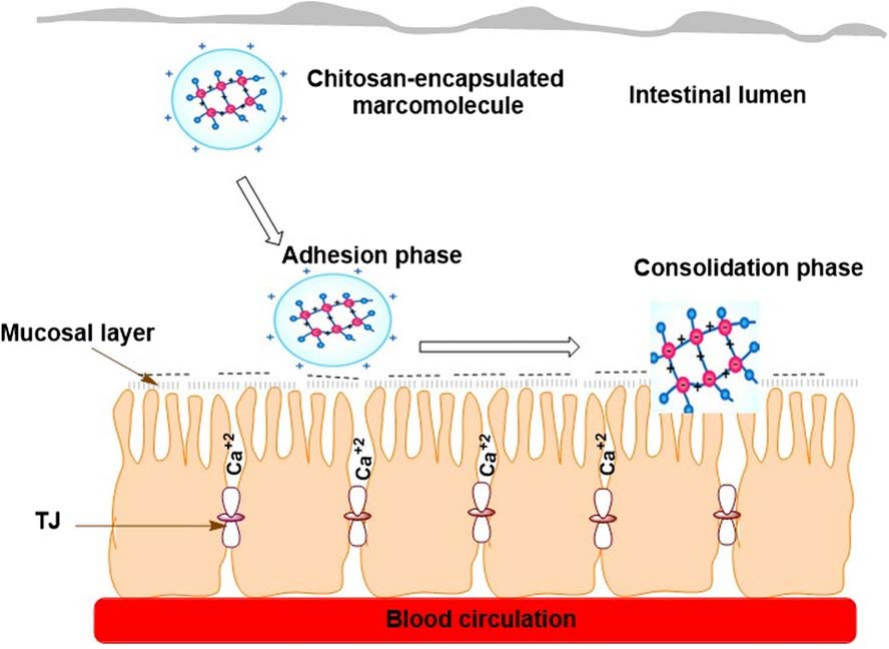
The attachment and consolidation stages of a positively charged mucoadhesive polymer

Bioadhesion is a circumstance resulting from the attractive forces generated between a polymer and the surface of biological substrates, which enables the polymer to tightly stick to the biological substrate for various periods of time, depending on the nature of the forces participating [67]. As regards the phenomenon of mucoadhesion, two phases can be distinguished: the adhesion phase between the polymer and the mucosa, which enables the polymer to diffuse and dilate, and the integration phase as a result of the development of different adhesion forces (Fig. 2) [67, 68].

To date, six hypotheses have been proposed to express the phenomena behind the two stages of mucoadhesion, which are:

- The electronic theory is based on the transfer of electrons amongst the polymer backbone and the substrate, leading to the development of binding forces.
- The wetting theory proposes the higher affinity of the surrounding liquid to substrate surface to the surrounding liquid medium resulting in case of lower angle of contact.
- The cohesive theory describes that bioadhesion phenomena are basically attributed to the interactions arising between similar molecules.
- The adsorption theory expects the existence of molecular attraction based on van der Waals or H-bonding between the surfaces of the biological substrate and the polymer.
- The diffusion hypothesis supposes the formation of a networked structure as a result of the polymer backbone spreading on the mucosal surface along the adherent interface.
- The mechanical hypothesis describes the adhesion developing between the substrate and the polymer as a result of the interlinking of the polymer’s structure with the micro-holes present on the biological surface [69].

The effectiveness of various drugs may be improved by applying mucoadhesive delivery systems, which stay in direct contact with the targeted mucosal surface, hence they release the incorporated macromolecule directly to the absorptive tissues, thus enhancing the delivery efficiency, and they can be used either for local or systemic effects. Therefore, mucoadhesive polymeric systems are attractive carriers for protein delivery as their properties may be tuned as a result of various changes in their network structure or swelling behavior as a response to various surrounding triggers, e.g. the change of pH, electric field, temperature, light or ionic strength [59]. In addition, they may isolate protein/peptide from the degradation effect of the low gastric pH as well as of proteolytic enzymes [70–72]. Moreover, they control the release of incorporated molecules from the delivery system and provide the concurrent release of the drug and the enzyme inhibitor. Furthermore, they also localize the effect of enzyme inhibitors as well as make the drug closer to the absorption site for a sufficient time [73]. This effectiveness was confirmed in recent studies, where mucoadhesive devices containing a mixture of polymers with mucoadhesive character were developed. These enteric coated devices were entirely coated with water impermeable backing layer except on one side, where the device will adhere to the intestinal mucosal membrane, making the release of incorporated macromolecules possible in a unidirectional pattern. These devices provide protection from luminal proteases; therefore, they prevent the loaded drug from enzymatic degradation. Moreover, the investigations showed that the developed devices are safe and can tolerate the shear stress of peristalsis due to strong mucoadhesion, and were reported as an efficient alternative to insulin injection in controlling diabetes [74, 75].

There are numerous available and commonly used mucoadhesive polymers including chitosan, carbopol, cellulose derivatives and alginate. Nevertheless, the selection of the polymer type and its molecular mass should be done very carefully before utilizing it in the formulation, as the release of the peptide may be retarded because of steric hindrance if polymers with a higher molecular weight are used [76]. Most of the polymers which exhibit the strongest interaction with mucins are hydrophilic and positively charged under the pH conditions of the GIT. Chitosan (poly [ß-(1–4)-2-amino-2-deoxy-D-glucopyranose]), a positively charged polymer derived by the partial deacetylation of chitin [77], was described to form a strong matrix with mucus glycoprotein, enabling it to release insulin and significantly controlling the plasma glucose levels in normal rats for 24 h [78]. In addition, chitosan can sustain drug release, extend the duration of drug treatment time and concurrently enhance the mucoadhesive force of drug particles to the mucosal membrane at the absorption site [79].

Chitosan and its derivatives have been utilized by many researchers for protein/peptide delivery particularly because, besides their mucoadhesive properties, they are recognized as effective and safe absorption enhancers, which considerably improves their capacity for the delivery of hydrophilic macromolecules through the (nasal and peroral) mucosa [80]. The mechanism of permeation enhancement is attributed to the free positive charges, enabling strong adhesion with the absorptive substrate and leading to the modulation of the TJ proteins [81]. As it was discussed above, the interaction between the mucosal membrane (mucin) and polymers is mostly based on non-covalent bonds, but some polymers are also able to form covalent bonds [82]. This novel class of mucoadhesive polymers, often called multifunctional polymers, have recently replaced conventional polymers on the market thanks to their distinctive attributes, such as considerably improved mucoadhesive characteristics and similarly improved permeation enhancing effects [83]. These novel polymers (like poly (acrylic acid)–homocysteine, chitosan–iminothiolane, chitosan–thioglycolic acid, poly (acrylic acid)–cysteine, chitosan–thioethylamidine, alginate–cysteine, poly (methacrylic acid)–cysteine and sodium carboxymethylcellulose–cysteine) have been produced by thiomerization. This improves their water uptake, which – along with intra- and interchain disulfide linkages – improves viscosity, strengthens cohesiveness and mucoadhesion, which are in turn responsible for the prolonged residence of the polymeric system at mucosal surfaces [84, 85]. The newly developed preactivated polysulfonate thiomers also showed a distinct improvement in the paracellular transport of both low and high molecular weight hydrophilic penetration markers along the monolayer cells of Caco-2 of newly enucleated rat gut; therefore thiolated polymers are recommended as potential carriers, particularly for orally administered macromolecules [86].

### Prodrug approach

Prodrugs are inactive forms of therapeutic molecules produced via the chemical modification of the original molecule, which turns into the active form of the molecule during administration, commonly by the effect of enzymatic reactions or other possible reactions inside the body [7]. The primary goals of the prodrug approach can be outlined as follows: targeted release, ameliorating absorption or membrane permeability and decreasing metabolism or side effects [87]. The generation of prodrugs from proteins/peptides appears to be an attractive approach concerning the improvement and optimization of their delivery because all the basic objectives of this approach may be fulfilled with the modification of the structure of biopharmaceuticals [88]. These hydrophilic molecules require a certain increase in lipophilicity to penetrate the epithelial cell membrane and thus to cross the cells [89]. Chemical alteration on a reactive amino acid like lysine and cysteine or other amino acids will not only give rise to sustained absorption and reduce the amount of drug required to produce the therapeutic effect but will also improve stability as well as decrease immunogenicity [90, 91], and based on the size of the conjugated molecule renal ultrafiltration may be decreased due to the increased molecular size of the polypeptide [92]. The nature of the conjugated molecule may be varied in a wide range such as direct modification by the use of acetylation, C-amidation, N-pyroglutamate conjugation, PEGylation (PEG) or glycosylation [93], or via the sugar part of the glycoprotein [13]. Other strategies used for prodrug formation include d-amino acid substitution, olefenic substitution, carboxyl reduction, dehydro-amino acid substitution, retro inversion modification and thiomethylene modification [94]. Substantial success was achieved in producing protein prodrugs, but due to the structural complexity of proteins, this tool was unsatisfactory when attempting to modify most proteins, and successful modification also faced the problem of overall low yield [95].

#### Mimetic peptides approach

Parallel peptides are peptides with abnormal arrangement of synthesized amino acids or incorporation of different new linking bonds between those amino acids. The inclusion of these chemical changes provides the preservation of peptides against peptidases, which have high specificity towards normal peptides, but the main drawback of this approach is the change or loss of the biological activity which should be retained as the initial one [96].

#### Fatty acids-conjugation (lipidation) approach

Lipidation is the chemical alteration of a hydrophilic biomolecule, made by the addition of a lipophilic entity mainly via the acylation reaction to improve both the delivery and the pharmacological efficiency of macromolecular drugs by influencing membrane transport, metabolic stability and bioavailability [97]. The covalent modification of proteins can be done with various lipophilic substances, including isoprenoids, lipid acids and fats. Accordingly, the lipidation process has a great role in tailoring as well as in localizing proteins [98]. Large numbers of proteins, including many proteins utilized in the therapy of human diseases, are modified by covalently linking fatty acids and/or isoprenoid groups, which play a basic role in regulating their structure and function. Palmitate and myristate are the two fatty acids most commonly linked to proteins [99]. Reversible lipidation represents an effective way to retain the basic biological activity of the lipidized molecule. Lately, a reversible lipidation method has been accomplished to guarantee the re-formation of the therapeutic peptide from its lipidized form subsequent to oral absorption [89].

#### Cell penetration peptide (CPP) conjugation approach

CPP is a peptide with a high penetration capacity across the absorptive cell membranes, thus a conjugation of the CPP to macromolecular drugs like proteins will improve their kinetics. Furthermore, a macromolecule and CPP can be administered as a simple mixture [100]. At present, various non-injection routes including nasal, pulmonary and oral routes have been developed by utilizing a conjugate of CPP with antidiabetic peptide for controlling blood glucose level. Most of such research reported that after a suitable CPP conjugation with antidiabetic peptide hypoglycemic activity could be retained. Furthermore, they show better stability and resistance to proteolytic degradation [101].

#### Protein-polymer conjugation

In comparison with lipidation, the covalent conjugation of proteins with various polymers offers the advantage of the wider range of the targetable side chains, which results in altered solubility, lipophilicity, targetability, crystallinity and taste. Consequently, pharmaceutical and biotechnological companies are conducting numerous studies and testing new techniques to find the ideal modification [97]. Short chains of both chitosan and polyethylene glycol (PEG) represent the most utilized conjugates because they overcome the issue of low solubility and improve the formulation stability in the GIT [102]. Lee et al. developed a conjugate of insulin and low molecular weight chitosan (LMWC) in an attempt to enhance the oral delivery of insulin. The conjugates were found to have good ability to manage the plasma glucose level for several hours in diabetic rat models, and they are considered as a potential future technique for improving the efficacy of orally administered therapeutic peptides and proteins [103]. Moreover, a conjugate of insulin and low relative molecular mass protamine as CPP has been incorporated into mucoadhesive nanoparticles (MNPs), and the composite showed an effective delivery of insulin following oral application. MNPs were found to render the loaded conjugates in direct contact with the intestinal absorptive tissues. As a result of their high permeation, it is possible for the released conjugates to be absorbed without digestion, and hence higher bioavailability of insulin in diabetic rats has been obtained [104].

### Lipid-based drug delivery system (LBDDS) approach

Lipid excipients are commonly involved in a formulation to increase the absorption of drug molecules along the intestines by different mechanisms, including limiting intestinally mediated proteolysis, increasing membrane permeability and enhancing intestinal lymphatic uptake [105]. Therefore an emerging interest was observed concerning LBDDSs over the past two decades despite the pharmaceutical difficulties entailed by these candidates [106]. Lipid-based carrier systems as drug vehicles are composed of physiological lipids and offer several advantages, including high biocompatibility and controlled release based on the nature of natural lipids, no susceptibility to erosion phenomena compared to polymeric systems, easy and simple manufacturing by compressing or moulding, and slow water uptake after administration, which may offer a less damaging environment for the loaded proteins [107]. Thus, they have most of the advantages without the risks and regulatory concerns involved in the direct conjugation of proteins with lipids.

#### Liposomal encapsulation approach

Liposomes are defined as microscopic vesicles with a spherical shape, consisting of two compartments, an inner aqueous sinus surrounded by one or multiple homocentric lipid bilayers. The liposomal membrane consists of reasonably biocompatible, biodegradable and non-immunogenic natural and/or synthetic lipids usually stabilized with cholesterol, which also extends the circulating time [108, 109]. The versatile nature of liposomes enables lipophilic drugs to be incorporated within the lipid bilayers, while lipophobic molecules like proteins may be solubilized inside the internal aqueous core [110]. Therefore liposomal carriers were utilized for the successful encapsulation of various therapeutic molecules like tropicamide, artemether, paclitaxel, acyclovir, cyclosporine, dithranol and chloroquine diphosphate [111]. In addition, they represent excellent carriers for the delivery of protein antigens as they may be functionalized to mimic pathogens, which may induce the immune system due to their enhanced uptake by antigen presenting cells through various mechanisms, and the increased exposure of liposome encapsulated antigens to the lymphocytes of the immune system [112]. In recent years, several liposome-based vaccines have been designed to deliver oral antibodies to target several diseases caused by viruses and bacteria, such as *Salmonella enteritidis* and influenza-A viral vaccines. Thus, liposomes have shown high capacities to deliver various antigens, such as peptides/proteins and DNA [113].

Compared to various lipid carries, liposomes have high capacity to enclose and protect labile molecules against the hazardous GIT environment which would result in denaturation, and they may also increase absorption into enterocytes via the stimulation of their chylomicron production, thus promoting drug transport [114]. Protein drugs of interest may be both enclosed inside the liposomes or chemically attached to the outer surface of the vesicles. The simple enclosure of a macromolecule can be attained by the incubation of a macromolecular drug alongside the vesicles at or somewhat below the transformation temperature of the constituting lipids, whereas triggered (active) loading of biopharmaceuticals can be achieved by the gentle swirling of liposomes in the presence of a buffered alcoholic solution of the proteins at elevated temperature for a specified period of time [115].

Despite their numerous advantages, liposomes pose considerable issues regarding physical, chemical and biological stability, and these issues should be investigated and evaluated thoroughly in the course of research, during and after preparation to achieve a good background stability profile. Similarly, the development of general guidelines for the stability testing of liposomes would also be necessary [116]. The chemical stability of lipids against hydrolysis or in the case of unsaturated lipid chains also against oxidation is a point of concern, especially during the storage period. Therefore, it is recommended to store liposomes frozen or in a lyophilized powder form, but in this case the re-check of their size distribution, drug load and morphology before use is essential [117]. Furthermore, the development of liposomal protein delivery systems has to face other challenges as well, such as low protein loading efficiency, especially when using a small vesicle size (range of 50~150 nm), or the instability of the encapsulated protein during preparation, particularly under harsh processing conditions or when using organic solvents [118]. Overall, numerous issues such as the presence of organic solvent residues, physical and chemical instabilities, sterilization and pyrogen control (when designed as injectable), variation in size distribution, difficulties in batch to batch reproducibility and shortened half-life due to pancreatic lipase and bile salts should be overcome during the formulation of liposomes. This explains why only a limited number of liposome-based drug formulations for oral delivery may be found on the market today [119, 120]. A further issue is that liposomes designed to tolerate the harsh GI environment may exhibit decreased permeability across GIT epithelia, which constitute the main barrier to absorption [121]. However, the rational design approach to attain therapeutic goals might represent the rate-determining step in the development of more advanced liposome-based oral therapeutics in the future [122].

#### Solid lipid nanoparticles (SLNs)

To overcome the previously discussed drawbacks of liposomes, two different research groups have developed SLNs loaded with insulin for application via the oral route [123, 124]. SLNs are nanosized lipid carriers with particle sizes of 50–1000 nm, which remain solid at ambient and body temperatures. SLNs usually contain physiological lipids, for example, glyceride mixtures and steroids. They are stabilized by biocompatible surfactants and represent an alternative to liposomes and other nanoparticles [35, 125]. These loaded SLN formulations exhibited good efficiency to improve the gastrointestinal absorption of insulin, which was confirmed by the plasma sugar level of the tested rats, which was lower than that of the rats receiving oral insulin solution and unloaded SLNs (control) for one day. Accordingly, loaded SLNs showed a partial protection of insulin against luminal proteases, therefore they are considered as stable carriers to deliver oral insulin with good results of controlling plasma glucose level [123, 124].

SLNs are increasingly used as the protective delivery systems of labile drugs as well as to control/sustain the release of incorporated molecules due to their low toxicity and superior physical stability compared to other lipid-based carrier systems [126]. In addition, SLNs may have excellent reproducibility even with the use of various organic solvent-free methods. Besides their relatively easy manufacturing, SLNs may positively affect drug uptake through various ways, such as enhancing the extent of solubility, hindering drug precipitation upon dilution, suppressing efflux transporters, increasing both membrane permeability and lymphatic uptake. Nevertheless, despite the numerous advantages, the low loading efficiency, particularly for hydrophilic drugs, and the possible expulsion of drugs after polymeric transition during storage still pose considerable problems to scientists [127, 128]. In spite of these drawbacks, their flexibility in preparation and the simplicity of large-scale production may encourage the widespread use of SLNs [129].

Further enhancement of the orally administered medicinal proteins may be accomplished if the lipid-based nanocarriers are conjugated with polymers. In a recent study, poly lactic-co-glycolic acid (PLGA)–lipid lipospheres were developed, which consisted of a PLGA lipophilic interior and a self-assembled lipophilic layer at the interface. These lipospheres demonstrated high crossing efficiency along the microfold cells (an in vivo model), resulting in the efficient improvement of the intestinal absorption for the loaded protein molecules over regular polymeric nanoparticles [130]. Another research group recently developed and formulated low molecular weight (LMW) chitosan-lipid nanoparticle composites to deliver siRNA into the cytoplasm. The formulation gave promising results as it takes benefit of the mucoadhesive and permeation-enhancing properties of chitosan as well as utilizes the hydrophobic reservoir capacity of the hydrophobic core of the hybrid particles [131].

### Polymeric nanoparticles approach

From the pharmaceutical aspect, both polymeric micro- and nanoparticles are of emerging interest since they show better stability and therefore better preservation capacity against the degrading effect of the GI environment compared to the carriers of fatty origin, such as liposomes [132]. The effectiveness of protein drugs may be improved successfully with both micro- and nanoencapsulation [133] via protection from hydrolysis and proteolytic enzymes and the improvement of their absorption, in addition to their mucoadhesive properties and permeation enhancing characteristics [134]. However, it was observed that while microparticles are absorbed only through the microfold cells, nanoparticles may also utilize the same pathway and are also able to penetrate cell membranes, therefore the quantity of nanosized carriers penetrating through the intestinal membrane is higher compared to microspheres [134]. The penetration and absorption enhancement properties may be further improved by tailoring its surface characteristics to adjust mucoadhesion property, lymphatic and cellular uptake and site-specific absorption. Nanoparticles are derived mainly from the most common polymers, like poly (lactic-co-glycolic acid) PLGA, poly lactic acid (PLA) and poly sebacic acid (PSA). These polymers perform their mucoadhesivity through different possible methods of interactions, such as covalent and non-covalent bonding or the involvement of both [135]. From this aspect, especially chitosan and its derivatives showed high safety, biocompatibility, biodegradability and represent multifunctional polymers since besides their extremely good mucoadhesive properties, their penetration enhancing effect was also reported [133]. Because of this multifunctionality, chitosan NPs are promising drug delivery carriers suitable for a wide group of drugs, including labile drugs and macromolecules [136, 137]**.** The combination of various polymers and the utilization of oppositely charged polyelectrolytes like chitosan, polyacrylic acid, alginate, polyalkylamine hydrochloride in the formulation of layer by layer (LBL) coated nanoparticles may also offer further improvement and show a great impact on both macromolecular drug stability and the oral absorbability of protein from the GIT [138]. All in all, mucoadhesive polymer nanoparticles were successfully used for the delivery of the extracellular products (ECPs) of *Vibrio anguillarum* to deliver oral vaccination in turbots [139], where it was confirmed that the process of insulin uptake seemed to be a joint process of both insulin crossing the intestinal cells and the uptake of the insulin loaded nanoparticles by aggregated lymphoid nodules. However, Yao et al. showed the main limitations of nanoparticulate carrier systems are usually associated with limited loading efficiency and particle agglomeration due to thermodynamic instability [140], which was also observed by Gao et al., who found that the efficiency of loading was only 57.8 ± 2.54% for the turbot vaccination [139].

### Self-assembling bubbles carrier approach

Besides the previously discussed approaches, recent studies by the research group of Chuang E-Y introduced a very innovative bubble carrier system as delivery vehicle for the oral delivery of insulin, with possible application as a technique for the oral application of other medicinal macromolecules [141]. This self-assembling bubble carrier composed of pentetic acid, carbonate, surface active agent and insulin was enclosed in oral capsules and enterically coated to bypass gastric acidity. Once the formulation reaches the lower part of GIT fluids, it breaks down and releases acid and bicarbonate, which react quickly and produce carbon dioxide, which acts as a transporter for the involved insulin. The obvious elevation in plasma insulin level accompanied with a decrease in plasma glucose level was noticed in diabetic rats. Accordingly, self-assembling bubble carriers represent an effective and safe method suitable to deliver other biologically active macromolecules [55].

## Conclusion

The oral delivery of biopharmaceuticals is a challenging research area as a result of many difficulties, for example, the rigid physical barriers of absorptive tissues for these high molecular mass, hydrophilic drugs, and the degradation by gastric juice and intestinal metabolizing enzymes, all together acting as pharmacokinetic barriers and are responsible for the absorption of a tiny amount of the orally administered dose. Accordingly, the first step in the formulation requires comprehensive knowledge about these barriers. For this reason, most attempts focused on overcoming the enzymatic barrier within the lumen and on improving the permeation of macromolecules.

This review reveals the versatility of methods and involved excipients to overcome the bioavailability problem. From industrial aspect, the combination of mucoadhesive polymers, EIs and PEs in a conventional dosage form appears to be the most applicable approach, as the concurrent release of these excipients may form appropriate microenvironment to obviate the protease barrier and achieve facilitated absorption of the loaded macromolecules. Especially if the effect is localized on the absorption site by the mucoadhesivity of the carrier which increases the chance for absorption. However, the incorporation of these agents may be critical and hence careful screening is required. Nevertheless, some molecules such as phenyl piperazine (0.1%*w*/w), SDS, chitosan and its derivatives were investigated recently and regarded as potential enhancers with reliable safety. The use of multifunctional excipients such as CA to inhibit enzymatic activity by the careful modification of the pH in which peptidases are more active at the targeted absorption site as well as to facilitate paracellular absorption by modulating the permeability of TJs due to the chelating of membrane-bound calcium may further increase the safety of the carrier.

PEGylation by attaching one or more PEG series was developed to increase the circulating time and hide the linked macromolecule from the enzymatic attack, but PEGylation and other prodrug models may conflict with the general regulatory rules and may require more intensive testing of the produced conjugate.

The utilization of lipid-based nanocarriers (e.g. liposomes or SLNs) for the delivery of macromolecules seems to be an efficient technique for oral administration as it provides protection and internalization through the stimulation of intestinal lipoprotein transporters and the possibility to encapsulate hydrophilic and hydrophobic molecules at the same time. On the other hand, the difficulty to reproduce in the same manner, physical and chemical instability during storage, low loading capacity and degradation by pancreatic lipase represent the main limitations and may restrict their utilization as oral macromolecule carriers. From the aspect of reproducibility, SLNs are better than liposomes. However, due to the lack of the hydrophilic interior, they provide poor drug loading capacity for hydrophilic macromolecules and hence, the expulsion of hydrophilic drugs was observed during storage, which considerably decreases the shelf-life of these products.

From the aspect of stability and loading capacity, mucoadhesive polymeric micro/nanocarriers may offer better solution compared to lipid nanocarriers, due to their more hydrophilic structure. However, despite their protecting effect against both luminal and mucosal secretions and enzymes their effectiveness in enhancement of therapeutic effect may be limited due to their lower internalization efficiency. Nevertheless, among them, chitosan micro/nanoparticles proved to be prospective drug delivery carriers as they offer many advantages, including safety, biocompatibility, biodegradability, micro/nanosized nature and the ability to open reversibly TJs, which may facilitate drug uptake through the cell membrane, while their mucoadhesive property increases the residence time at the site of absorption.

Accordingly, it can be concluded that formulation of hybridlipid/polymeric micro/nanoparticles would be the most appropriate carrier systems for the oral delivery of therapeutic macromolecules as it may provide appropriate loading capacity and stability with improved internalization capacity.
